# Polydopamine-based Implantable Multifunctional Nanocarpet for Highly Efficient Photothermal-chemo Therapy

**DOI:** 10.1038/s41598-019-39457-y

**Published:** 2019-02-27

**Authors:** Arjun Prasad Tiwari, Deval Prasad Bhattarai, Bikendra Maharjan, Sung Won Ko, Hak Yong Kim, Chan Hee Park, Cheol Sang Kim

**Affiliations:** 1 Division of Mechanical Design Engineering, Chonbuk National University, Jeonju, 561-756 Republic of Korea; 2 Department of BIN Convergence Technology, Chonbuk National University, Jeonju, 561-756 Republic of Korea; 3 Department of Bionanosystem Engineering, Graduate School, Chonbuk National University, Jeonju, 561-756 Republic of Korea; 4 Department of Chemistry, Amrit Campus, Tribhuvan University, Kathmandu, Nepal

## Abstract

We report a design and fabricate multifunctional localized platform for cancer therapy. Multiple stimuli-responsive polydopamine (PDA) was used for surface modification of electrospun doxorubicin hydrochloride (DOX) loaded polycaprolactone (PCL) fibers to make a designated platform. Photothermal properties such as photothermal performance and stability of the resulting composite mats were studied under the irradiation of the near-infrared (NIR) laser of 808 nm. With the incorporation of PDA into the fiber, a remarkable increase of local temperature was recorded under NIR illumination in a concentration-dependent manner with excellent stability. Drug released assay results revealed PDA coated PCL-DOX mats showed pH and NIR dual responsive behavior thereby exhibiting improved drug release in an acidic medium compared to physiological pH condition (pH 7.4) which is further increased by NIR exposure. The cancer activity *in vitro* of the mats was evaluated using cell counting (CCK) and live and dead cell assays. The combined effect of NIR mediated hyperthermia and chemo release resulting improved cells death has been reported. In summary, this study presents a major step forward towards a therapeutic model to cancer treatment utilizing pH and NIR dual responsive property from PDA alone in a fibrous mat.

## Introduction

Recently, regional drug delivery (RDD) is gaining much more interest in solid cancer treatment over its conventional drug delivery system. Nanoparticulate drug delivery system as a conventional drug delivery system encounters multiple challenges to complete tumor growth inhibition and eventual tumor eradication owing to rapid clearance, rapid uptake by the reticuloendothelial system and improper drug distribution^1,2^. To this end, RDD has been an iconic platform to modulate the drug on temporal basis matching the physiological needs while reducing the common toxicities associated with chemo drugs to the healthy tissues, thereby enhancing the therapeutic efficacy. Electrospun nanofibers are becoming a device of interest in drug delivery applications because of their versalities of easy fabrication, tunable nano/microstructures, controlled fabrication, improved drug loading, customizable mechanical properties and implantable properties^3–8^. However, controlled and precise drug delivery is a difficult issue from only drug-loaded fibers. For example, hydrophobic PCL or polyurethane (PU) fibers can allow the slow and prolonged drug release for up to months^9,10^. But for the cancer treatment, this cannot be good enough to control the tumor growth due to heterogeneous tumor structure and aggressive growth of tumor cells. The rationally controlled drug release from implantable delivery systems is essential to avoid excessive or deficient drug release and ensure a certain therapeutic drug concentration at the tumor site for an extended time. In these consequences, development of smart drug depot that can provide on-demand localized drug release would be a critical choice.

Cancer tissue offers different microenvironmental conditions compared to adjacent normal cells or tissues in terms of pH, oxygen level, and temperature^11^. Several groups have utilized these unique features to modulate the drug delivery in a precise manner to the aim of achieving control drug release and enhanced efficacy^4,5,12,13^. Stimuli-sensitive drug delivery systems are in the crucial drug administration as they can respond to small signs and changes in the adjacent environment. Wang group demonstrated that a mussel-inspired protein PDA coating can finely tailor the pH-responsive loading kinetics and release of charged molecules^13^. However, the release of drug in pH dependency only cannot be enough to control the tumor growth because tumor tissues physiological conditions are varied from the surface to core. Sometimes tumor develops the resistivity to chemo. Therefore, a combination of pH-responsive drug release with other therapeutic models such as radiotherapy in RDD, alternating magnetic field (AMF) induced hyperthermia, phototherapy, etc. have been applied to ensure destroying complete tumor cells^4,12,14–19^. Core-shell Cu9S5@mSiO2 nanoparticles, loaded with the DOX, were incorporated into PCL and gelatin nanofibers. When the composite fibers were implanted directly to the tumor site, enhanced orthotopic synergistic therapy was achieved by combined DOX release and photothermal effects from the Cu9S5 nanoparticles under λ = 980 nm laser irradiation^5^. We have also reported recently multifunctional platform of electrospun fibers loaded with anticancer drug and alternative magnetic field sensitive iron oxide for combinational anticancer therapy^6^. Results were appealing, combo model was found showing enhanced tumor cells deaths than to their individual therapeutic model. Among the strategies to introduce hyperthermia, photothermal therapy which utilizes the heat generated from laser irradiation of NIR light-absorbing agents to kill cancer cells, has been widely used and exhibited excellent combo therapeutic effects when applied in combination with chemotherapy. In hyperthermia induction purpose, usually, the inorganic compounds such as copper-based compounds^5^, iron oxides^4,6^, gold nanorods^20,21^, and lanthanide^12^ are commonly incorporated into polymeric membranes. These inorganic compounds can reside on the body for a longer time due to their non-biodegradation profile which can result in adverse side effects. Moreover, these have limitations for long time using and repetitive uses due to fragmenting or melting^22^. Most importantly, inorganic compounds hardly show a multiple stimulus responsive behaviors. Electrospinning of hyperthermia-induced inorganic particles in a polymeric solution is quite difficult due to their inherent aggregation^23^. Similarly, synthesis of such nanoparticles needs additional hard works. Therefore, it remains an ongoing anticipation to find candidates that are biocompatible organic compounds having multiple functionalities such as pH and/or NIR-triggered drug release and hyperthermia with excellent stability in a physiological condition that can combine in a drug-loaded fibrous matrix.

PDA is a biodegradable, biocompatible, and organic biopolymer which has been widely used in surface modification of biomedical devices due to its catechol group offering attachment to a wide variety of materials including polymers, metal oxides, and semiconductors^24^. Moreover, it shows multifunctional activities, such as pH and NIR-responsive behavior that is widely used in the biomedicine to successful triggering drug release and hyperthermia^13,25,26^. Mei group demonstrated PDA modified nanostructures containing anticancer drug can be used as a multifunctional co-delivery system for the targeted chemo, gene, and photothermal therapy against multidrug-resistant cancer^14,18,19,27,28^. NIR irradiation falls in the region of 650–1500 nm which has been widely used in the biomedical fields including tissue engineering and drug delivery because of safe compared to other light waves^29^. NIR triggered hyperthermia is found more user-friendly and effective to control the disease among other hyperthermia generating mechanism. Therefore, it would be a great effort to fabricate the drug-loaded fibrous meshes with multiple stimuli-responsive PDA coating to achieve a complete therapeutic window in a single platform. DOX was used as a model chemotherapeutic drug since it is broadly used to treat a variety of cancers alone or in combination with other treatment modalities. It works by inhibiting the synthesis of nucleic acids within cancer cells, thereby kills the cells^30^. PCL is taken as a polymeric membrane as a drug carrier since it has been extensively used in biomedical purposes owing to its excellent biocompatibility, tunable mechanical properties and slow biodegradation^31–34^. Although PDA has been widely applied in particulate drug delivery systems and hydrogels for photothermal therapy^14–19,24,35–38^, to the best of our knowledge the potential functional use of PDA in localized drug delivery using nanofibrous membranes has not yet been reported. The present study aims to develop smart multiple stimuli-responsive nanofibers that are capable of both pH/NIR dependent drug release in a cancer environment and heat generation under the application of NIR triggered phototherapy for the combined treatments of hyperthermia and chemotherapy.

## Experimental Section

### Materials

Poly (ε-caprolactone) (PCL, Mw = 70,000–90,000) was obtained from Sigma–Aldrich (USA). 2,2,2-Trifluoroethanol (TFE) was purchased from Alfa Aesar, USA. DOX.HCl was provided by Sigma, Korea. Dopamine hydrochloride was obtained from Sigma (Korea). Live and the Dead cell assay kit were purchased from Sigma, Korea. All other chemicals used were of analytical grade and were used as received.

### Fabrication of PCL-DOX mat

Electrospinning solution (20 g) was prepared by dissolving PCL and DOX (10 wt%) in TFE. The weight of DOX was set up 10% of PCL weight. The electrospinning solution was loaded into a 12-mL syringe. Syringe was then fitted onto syringe pump. During the electrospinning process, the solution was ejected from a syringe at a 1.5 mL/h flow rate with an applied voltage of 15 kV. The electrospun fibers were harvested on a polyethylene sheet that attached on the collector 15 cm away from the tip of the needle. Electrospinning was carried out in the chamber under stable conditions of temperature (T = 25 ± 1.5 °C), and relative humidity (30 ± 5%). Then as-prepared electrospun membranes were dried in a vacuum oven at 35 °C for 24 h. The mats obtained from electrospinning are referred to as PCL-DOX mat (PDP0). Similarly, pure PCL mat was also prepared under the same preparation conditions.

### Surface Modification of Fiber by PDA

**Figure 1:**
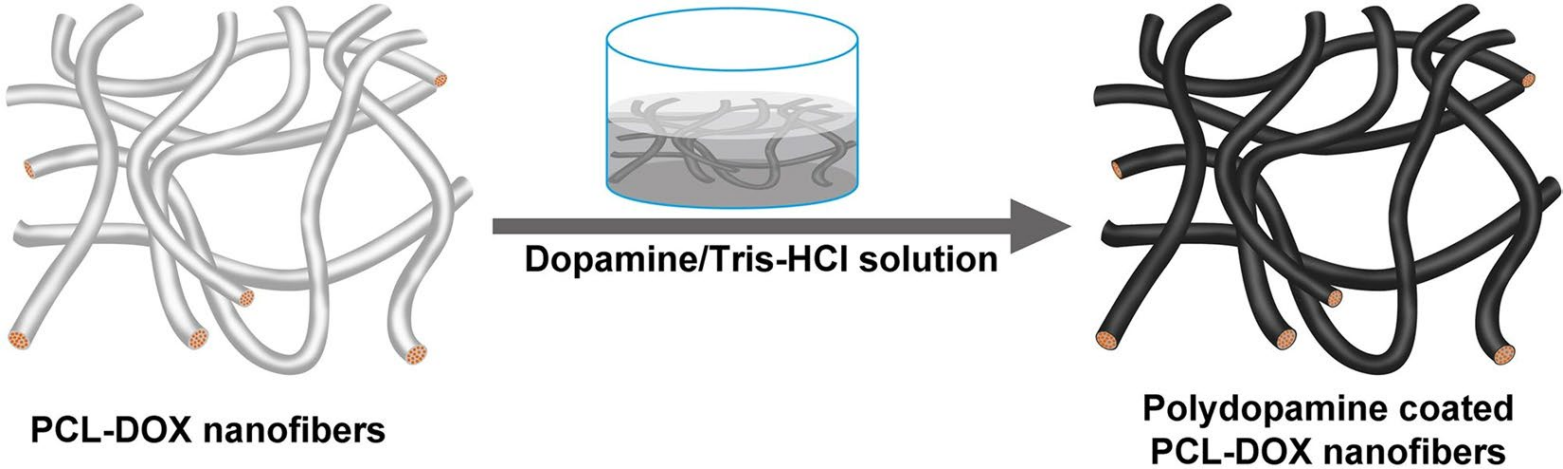
Schematic representation of the fabrication procedure of the PDA modified PCL-DOX nanofibrous mat.

Surface modification of the PCL-DOX mat (PDP0) by PDA is presented in Fig. 1. The desired size of the PDP0 mat was placed in a petridish containing 50 mL Tris buffer with different concentration (4, 8 and 12 mg/mL in 100 mM Tris.HCl) of dopamine monomer and allowed for 8 h at 4 °C for coating. Later, coated mats were washed thoroughly with deionized water and dried in a vacuum oven at 35 °C for 24 h. PDA decorated PCL-DOX nanofibrous mats obtained from 4, 8 and 12 mg/mL dopamine in tris buffer are referred to as PDP1, PDP2, and PDP3, respectively. Similarly, PDA coating was performed over pure PCL mat following the same condition as PDP2 fabricated and referred to as PP2 mat.

### Physicochemical Properties Study

Field emission scanning electron microscopy (FESEM, Hitachi SU-70, Japan) was used to investigate the structure and morphology of the as-spun PDA coated membranes. The average fiber diameter values from the SEM images were obtained from analyzing by Image J (NIH, USA) software. At least 100 nanofibers in different SEM images for each sample were analyzed to obtain the average fiber diameter. FT-IR experiments were performed at 25 °C on a PerkinElmer 580B infrared spectrophotometry over the spectral region of 400 cm^−1^ to 4,000 cm^−1^. Crystalline properties of the membranes were evaluated by an X-ray diffraction instrument (XRD, Rigaku X-ray diffractometer). Elemental identification of the PDA coated PCL-DOX mat was analyzed using an Axis Ultra DLD X-ray photoelectron spectrometer (XPS; Kratos, U.K.) equipped with monochromatic Al-Kα X-ray excitation source. Thermal gravimetric analysis (TGA, Q50 TA Instruments) was used to investigate the thermal stability of different mats; the samples were heated from 30 to 600 °C at a rate of 10 °C/min under a nitrogen purge of 40 mL min^−1^. Thermal analyses were performed using differential scanning calorimeter (DSC) (TMK 0017 universal analysis TA Instruments Co., USA). 8 mg weight of the sample was always used in DSC analyses. All DSC thermograms were taken under the nitrogen atmosphere. DSC scans were taken from −80 to 300 °C under nitrogen flow (50 mL/min) in all runs with scan rate at 10 °C/min (first scan). After the samples being quenched at −80 °C, second scans were made by exposing the samples upto 300 °C. To measure the mechanical properties of the mats, universal testing machine (AG-5000G, Shimadzu, Japan) with a 10 N load cell was used. For mechanical testing, mats were cut into dog-bone-shaped templates. The template was 40 mm length and 10 mm width. A cross-head speed of 5 mm/min was used for all tests. Thickness of mats was measured by digital caliper. Each sample was run to the point of failure and the ultimate tensile strength elongation at break and Young’s modulus values were obtained automatically.

### NIR-Responsive Photothermal Property Study

To study the photothermal effect of different mats, equal weight of samples placed on the 2 mL of PBS solutions were irradiated by a NIR laser (808 nm, 1.5 W/cm^2^) for 5 min with various power doses (1, 1.5 and 2 W/cm^2^), separately. The changes of the temperature on the mat was monitored in a real time by an infrared camera (FLIR C-Series). To investigate the photothermal stability of the membranes, two approaches were applied. Firstly, the temperature changes of the mats following ten heating (5 min at 1.5 W/cm^2^) and cooling cycles (5 min) were monitored. In another way, photothermal stability was studied by comparing the VIS-NIR spectra that linked to stability of the membranes. For that, samples were placed into chloroform for 4 h and subsequently incubated at 80 °C to remove PCL-DOX part. Thus, obtained PDA coating was washed, dried and minced to get 100 µg/mL suspension which was subjected to VIS-NIR analysis.

### Drug Release Study

Different mats each of 2 cm^2^ size were placed on the 15 mL tube containing PBS solutions with different pH 5.5, 6.8 and 7.4 and incubated for different time intervals at 37 °C in a shaking condition (120 rpm). At designated time points, whole release medium was collected and replaced with an equal volume of fresh PBS solution. UV–VIS spectrophotometry was used for measurement of the DOX concentration in the collected medium. NIR mediated release was assayed by applying the NIR into the mats that had been kept for different pH of PBS. Drug release experiments were performed three times. Obtained data were plotted as a cumulated release versus laser power (NIR) and time, separately.

### Cell Culture

Human lung cancer cells (A549) and a human breast cancer cell line (MCF7), were maintained as a monolayer culture in DMEM medium supplemented with 10% fetal bovine serum (Gibco) and 1% penicillin-streptomycin (Gibco) at 37 °C in a humidified atmosphere (5% CO_2_).

### *In Vitro* Localized Photothermal Cytotoxicity

The localized photothermal cytotoxicity of the PDA coated mats was evaluated using A549 and MCF7 cells with and without NIR laser illumination. Later, live/dead cell viability and CCK-8 assays were performed to monitor cytotoxicity according to our previous reports^7,39^. Membrane with 1 cm^2^ in size was attached to round coverslip, and placed into 48-well plate. Samples were ensured to sterilize by UV exposure for 2 h and subsequent washing with sterile PBS. Then, cells were seeded (50,000 cells/well) separately in the wells of a 48-well plates containing mats and allowed to incubate for 24 h. Cells were irradiated with NIR laser of varied intensities (1, 1.5 and 2 W/cm^2^) separately for 5 min. Cell viability was quantitatively measured another 24 h of photothermal treatment, using a CCK-8 assay according to the manufacturer’s instructions. Each experiment was performed in triplicate, and the data represent the mean ± standard error (n = 3). The untreated corresponding cells grown in tissue culture plate (48 well) were taken as control with 100% viability. We have further evaluated the IC50 values of different fibrous mats for MCF7 cells in designated condition. The drug concentration at which the growth of 50% cells was inhibited (IC50) in comparison with that of the control sample was calculated by curve fitting of the cell viability data^19,40^. Similarly, live and dead cell assay was performed to support the CCK-8 data. For the live and dead assay, MCF7 cells were washed three times with PBS, followed by staining with resazurin and SYTOX green according to the manufacturer’s protocol. Stained samples were observed by confocal microscopy.

### Statistical Analysis

All data are presented as a mean ± standard error (n = 3) unless otherwise mentioned. One-way analysis of variance (ANOVA) was used to analyze the significance of the difference between groups. P value less than 0.05 is considered statistically significant.

## Results and Discussion

### Fabrication and Characterization of PDA Coated Mats

**Figure 2:**
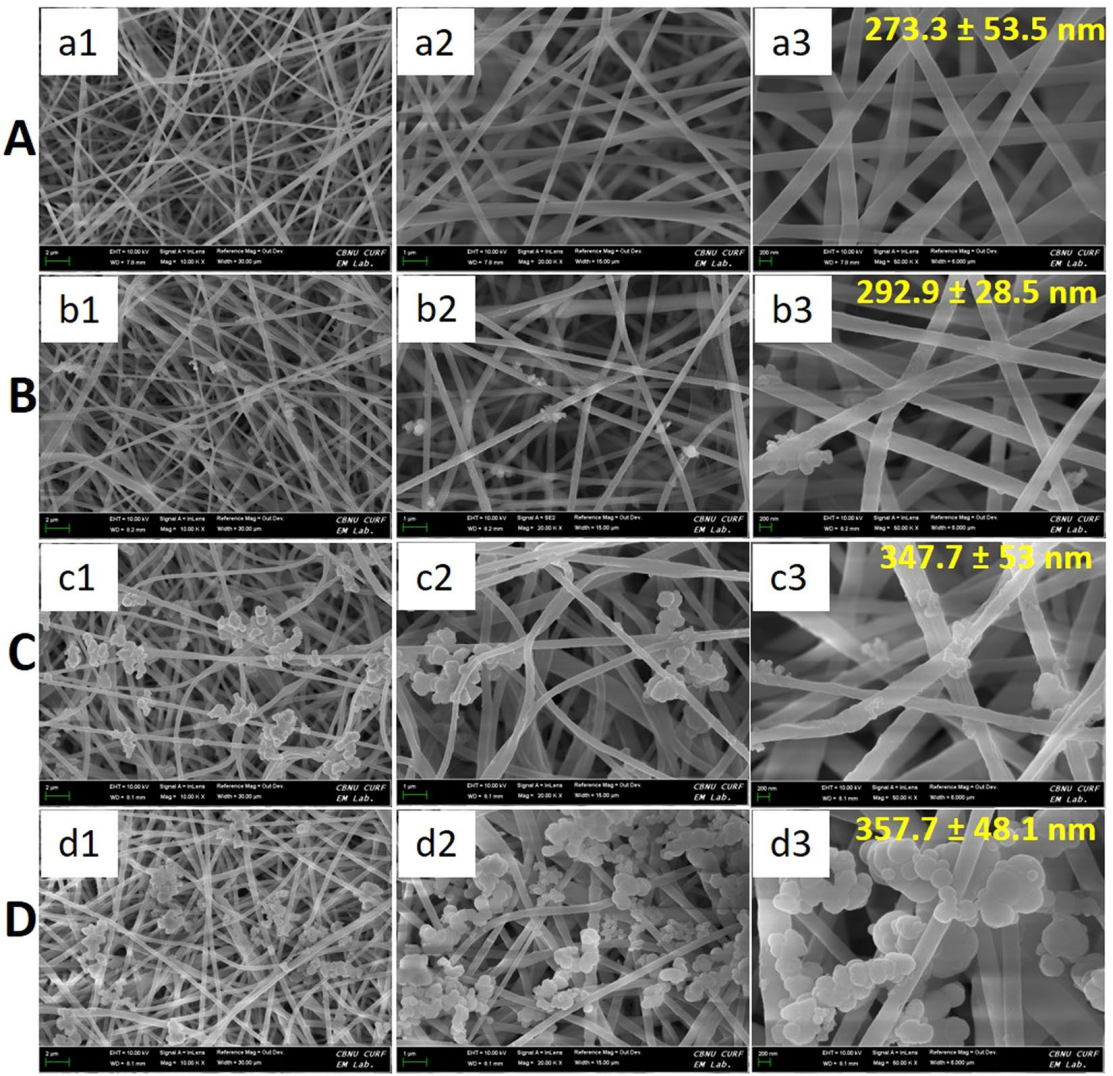
FESEM images of different mats at different magnifications. PDP0 (panel A), PDP1 (panel B), PDP2 (panel C) and PDP3 (panel D).

In this work, we chose the PCL as a model polymer to make an electrospun fiber, a drug carrier, due to its abundant uses in biomedical applications including drug delivery. PCL is a biocompatible, biodegradable, and non-immunogenic polymer with excellent mechanical properties^31,41,42^. Electrospun fibrous meshes oversee to use as a drug depot due to their high loading efficiency and allowing controlled and prolonged drug release. Firstly, PCL and DOX were blended and electrospun. Resulting PCL-DOX fibers are uniform in diameters without any secondary structures as confirmed by FESEM (Fig. 2A). The average fiber diameter is found to be 273.3 ± 53.5 nm.

**Figure 3:**
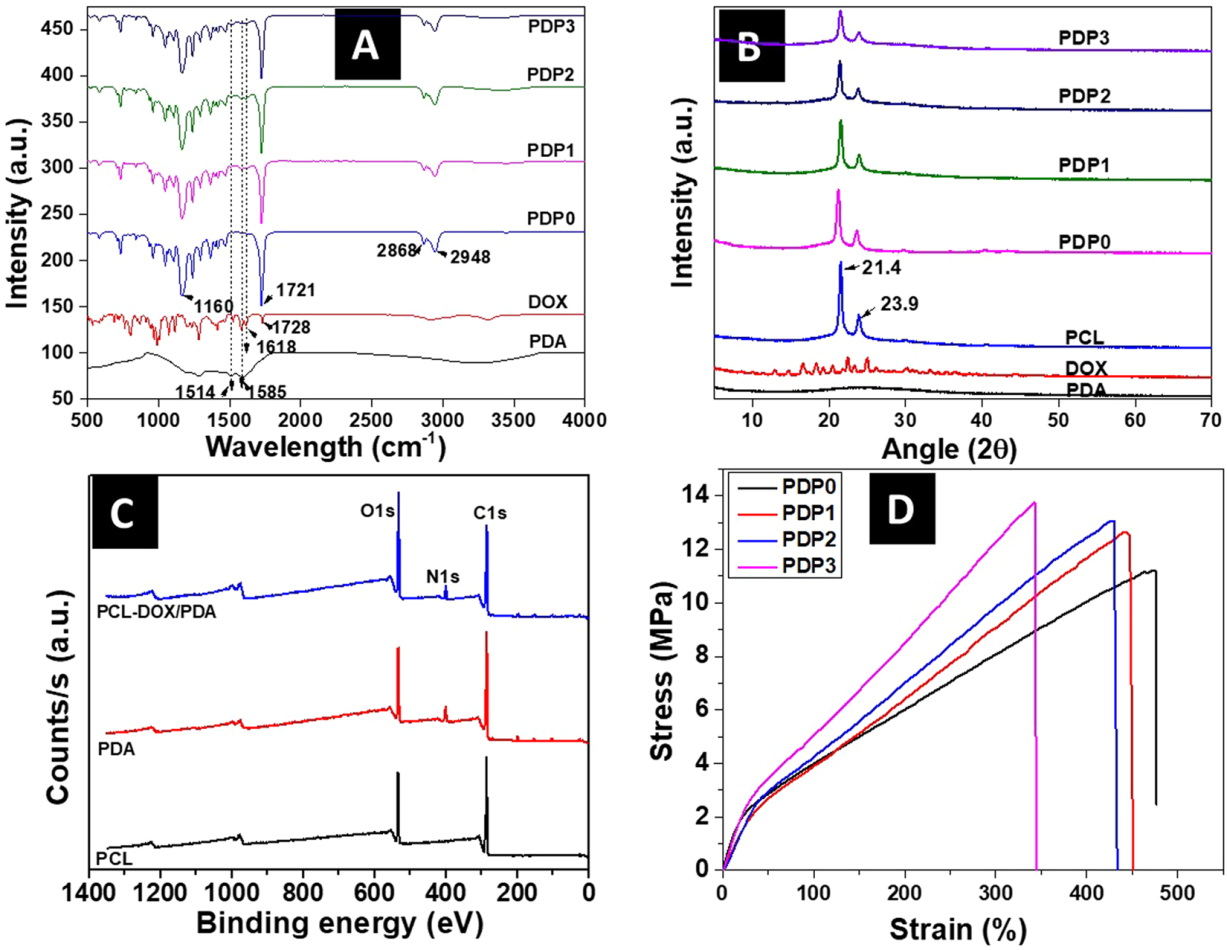
(**A**) FT-IR spectra, (**B**) XRD patterns, (**C**) XPS spectra and (**D**) Stress-strain curves of different samples.

To grab the abundant possibilities of PDA coating in order to make a multifunctional platform, PDP0 fibers were coated with various concentration of PDA by the chemical polymerization of dopamine. The PDA coated PDP0 meshes obtained from the polymerization of dopamine of 4, 8, and 12 mg/mL concentration are named as PDP1, PDP2, and PDP3, respectively. Figure 2 shows the influence of the concentration of dopamine on the surface morphology of the fibers. Each of the PDA coated meshes exhibited consistent fiber diameters (Fig. 2(B–D)). However, Fiber diameters are observed gradual increased with the increased concentration of dopamine. Average fiber diameter was found increased from 273.3 ± 53.5 nm of PDP0 to 292.9 ± 28.5, 347.7 ± 53.0, 357.7 ± 48.1 nm for PDP1, PDP2 and PDP3, respectively. In addition of a thin coating of PDA layer, some nodules like PDA particle- aggregates appear higher extent in PDP3 (Fig. 2D) compared to PDP2 (Fig. 2C) and PDP1 (Fig. 2B). The successful entrapment of DOX and coating of the PDA on PCL fibers was verified by FT-IR measurement (Fig. 3A). A new peak assigned to the DOX appears at the 1618 cm^−1^ (C=C band)^43^ in composites, confirms the loading of DOX into PCL fibers. Moreover, 1728 cm^−1^ (C=O band)^43^ for DOX is shifted to lower band at 1721 cm^−1^, can be related to the adsorption of DOX on the PCL. Moreover, most of the peaks associated with DOX are becoming reduced or disappeared possibly due to overlapping of the carrier polymer PCL or coating agent, PDA (Fig. 3A). Similarly, after modification of the PDA layer on the fiber surface, additional peaks assigned to PDA appears at 1585 cm^−1^ (C=C stretching mode)^44^, confirming the coating of a PDA layer on PDP0 fibers. Absorption bands at 2948 and 2868 cm^−1^ (stretching bands of CH_2_ groups), 1721 cm^−1^ (stretching band of the ester group C=O), and 1163 cm^−1^ (asymmetric and symmetric stretching of C–O–C) are associated with the PCL in PDP0 sample^31,45^. An apparent reduction in the intensities of all peaks corresponding to the PDP0 matrix were observed in a concentration-dependent manner once coating with PDA (Fig. 3A). Figure 3B shows the XRD spectra of different samples. PDA did not show any crystalline peak, suggests it is amorphous in nature. PCL exhibited the typical XRD peaks at 21.4 and 23.9° (Fig. 3B) revealing its crystalline nature, consistent with other reports^46^. Loading of DOX into the PCL fibers and/or subsequent modification of PCL fiber by PDA coating has not changed the peaks position, suggesting there was almost no change to the crystalline region in the PCL chain. Pure DOX shows its crystalline nature, however, typical peaks of DOX were disappeared in PDP0 fibers. This may be due to the entrapment of the DOX into the matrix to a much lesser extent compared to PCL which is consistent to others works^8,47^.

XPS was used to confirm the surface modification of PCL-DOX by PDA coating. The XPS spectra of pure PCL, pure PDA, and PCL-DOX/PDA are shown in Fig. 3C and Table 1. XPS spectra of PCL-DOX/PDA reveal the presence of all spectra corresponding to PCL (O1s and C1s peak BE at 533.95 and 286.27 eV, respectively) and PDA (O1s, N1s and C1s peak BE at 533.37, 401.03 and 286.12 eV respectively). It is worth noting that the elemental analysis of composite mat reveals the increase of the atomic percentage of oxygen and decrease of nitrogen as compared to PDA. Meantime, a decrease of carbon content was noticed for composite mat as compared to PCL and PDA (Table 1). These results are due to the coating of PDA layer over the PCL-DOX mat^15,16,48,49^.

**Table 1:** XPS spectra of different samples.

Materials	Elements with their peak binding energy (BE) and atomic percentages.					
	O1s		N1s		C1s	
	Peak BE (eV)	At %	Peak BE (eV)	At %	Peak BE (eV)	At %
PCL	533.95	21.14	—	—	286.27	78.08
PDA	533.37	19.09	401.03	7.24	286.12	69.99
PCL-DOX/PDA	532.70	26.52	400.40	5.98	285.85	65.02

### Mechanical Properties Study

Mechanical property plays a key role behind the success of any localized therapeutic platform, as forces exerted on the fibers may result in permanent deformation or even failure during the service time of the fibers. Herein, mechanical properties were measured using the mechanical tensile tester. The stress-strain curves of the different fibrous membranes are shown in Fig. 3D. Table 2 presents the mechanical properties including Young’s modulus, tensile strength, and elongation at breaks (%). Data reveal the tensile strength of PDP0 is increased from 11.2 MPa gradually to 12.6, 13 and 13.6 MPa for the PDP1, PDP2 and PDP3, respectively (Fig. 3D and Table 2), indicating stronger materials formed after modification with PDA. However, the strain of PDP0 mat was observed in opposite trend of stress when coated with PDA (Fig. 3D). The attachment of the PDA as a film reduced the mobility of the polymeric chains by improving interconnection among the fibers, thereby reduced the elasticity of PDP0 fiber^50^. However, same property of PDA may result in the improved tensile properties indicated by toughness in PDA modified fibers. PDP3 exhibited the higher Young’s modulus compared to the PDP0 mat (Table 2), suggests the increased stiffness than that of PCL. However, PDP1 and PDP2 have shown lesser Young’s modulus than the PDP0 mat. Results in the average revealed deposition of the PDA on to the drug-loaded mat can show the reinforcement effect. Tensile strength more than 10 MPa and Young’s modulus more than 6.5 MPa with appropriate elasticity can be good enough to support the soft and cartilages types of tissues^51^.

**Table 2:** Mechanical properties of the samples.

Materials	Elongation at break (%)	Young’s modulus (MPa)	Tensile strength (MPa)
PDP0	477.1 ± 27.1	10.4 ± 1.9	11.2 ± 1.9
PDP1	450.2 ± 29.3	7.3 ± 0.8	12.6 ± 0.9
PDP2	434.3 ± 28.5	6.4 ± 1.3	13.0 ± 1.5
PDP3	343.3 ± 37.4	10.1 ± 1.3	13.6 ± 1.3

Note: Each sample was evaluated in triplicate. The mean value and corresponding standard deviation are presented.

### Thermal Behavior Study

**Figure 4:**
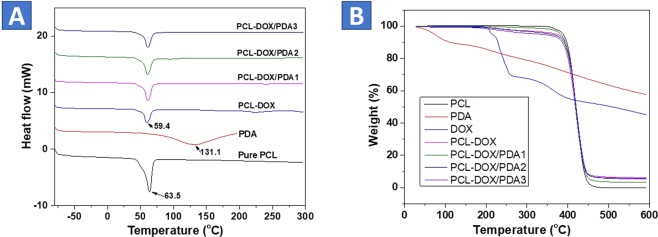
Thermal behavior of samples. (**A**) DSC and (**B**) TGA curves.

Thermal behavior of the samples was studied by DSC and TGA to understand the thermal behavior (Fig. 4). Figure 4A represents a DSC data. DSC data of PCL showed the endothermic peak at around 63 °C, is attributed to the semicrystalline PCL. With the incorporation of DOX into PCL fibers, slightly blue shifting of the peak (59.4 °C) occurred, is associated with the decreasing crystallinity of PCL fibers. The PCL and DOX are incompatible in solubility issue, therefore, the heterogeneous distribution of the DOX molecules into PCL matrix could result in the reduced crystallinity of PDP0 fiber^52^. A broad peak is observed for PDA as shown in Fig. 4A, represents its amorphous form. However, Further grafting of the PDA layer an endothermic peak (59.4 °C) of PDP0 matrix was shifted to slightly higher temperature  (61.2 °C), can be explained by slightly increasing crystallinity. The formation of transcrystalline features occurred in interfaces of PDA and PDP0 fibers can improve the crystallinity of the polymer^53^. However, the concentration of PDA coating was observed independently to changes of crystallinity. Figure 4B shows the TGA curves of different mats obtained after surface modification of PDP0 fibers. The figure shows only one major weight-loss stage was observed for pure PCL starting decomposition from 356 °C to complete annihilation at 427 °C. All samples lost some weight below 200 °C, is attributed to the moisture and solvent evaporation. Pure DOX showed four phase degradations. Only 55% was observed degradation heating up to 600 °C.

The Addition of the DOX into PCL exhibited obviously increased degradation temperature (Fig. 4B). Similarly, PDA shows 3 transitions for degradation, first degradation about 13% mass loss was occurred up to 179 °C, another 8% was degraded heating up to 324 °C and another major loss about 22% was observed after 324 °C. The significant mass loss in the last transition is associated with the degradation of the main PDA chain^54^. It is worth noting that mass residue was high as 57%. The PDP2mats exhibited decreased weight loss (residue 5%) at the temperature range compared to zero residues for the PCL fibers, indicating the loading of DOX and deposition of PDA layer could result in increased thermostability of PCL fibers. Because of the high thermal stability of PDA, the PDA film can protect the PCL-DOX fiber from being degraded, thus increases the degrading temperature.

### NIR-Responsive Photothermal Property Study

**Figure 5:**
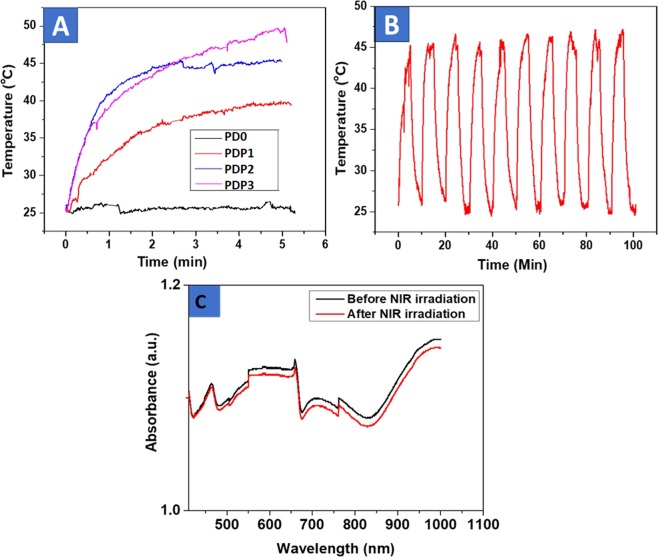
NIR-responsive Property Tests (**A**) Heating curves of different PDP2 mats, (**B**) Stability test; changes of the temperature profiles of PDP2 mat (PDP2) as a function of NIR on-off cycles (NIR on time: 5 min; NIR off time: 5 min) and (**C**) UV-VIS-NIR spectra of PDP2 before and after cycles of NIR irradiation. In this experiment, NIR laser of 808 nm wavelength with 1.5 W/cm^2^ power density was irradiated.

Since our motive is to evaluate the photothermal performance of the resulting composite membranes, it is important to understand their photothermal conversion capability and stability under the NIR irradiation. Photothermal properties were monitored based on the temperature elevation under NIR irradiation by FLIR camera. The temperature of composite mats PDP1, PDP2 and PDP3 were found increases by 13.7, 20.4 and 24.9 °C, respectively, after exposure to a NIR laser of 1.5 W/cm^2^ for 5 min while no apparent increases of the temperature of the PDP0 mat was recorded i.e. stays between 25–26 °C (Fig. 5A). Moreover, the gradual increases of the temperature under NIR were observed with increasing time of exposure (Fig. 5A). These data indicate the PDA coating of drug-loaded fibers increases the photothermal conversion capability in the PDA concentration and time-dependent manner. The photothermal conversion ability of the PDA coated fibers is related to the strong NIR absorbing capability of PDA, which has been reported by many groups^25,26^. Based on the temperature profile fibers had shown, PDP2 was selected for proceeding experiments because for a hyperthermia treatment temperature range of 42–46 °C is reported as an appropriate. In this temperature ranges, cells are undergone to apoptosis while higher than that ranges leading cell deaths via necrosis^55^. Since inadequacy to kill the tumor cells owing to their aggressive nature and chemo resistance of tumor cells, repeated treatment is needed, herein, the evaluation of photothermal stability is a crucial parameter for therapy. The mat was undergone on-off of NIR irradiation for 10 cycles (5 min on followed by 5 min off) and temperature elevation was checked each time (Fig. 5B). Results showed maximum temperature level remained consistent about 46 °C in each exposure time while it shows temperature fall down consistently under NIR off mode, indicating excellent stability of PDA coated PCL/DOX fiber under NIR. Furthermore, stability of the membranes was studied by UV-VIS-NIR spectroscopy. VIS-NIR spectra were taken before and after the membranes undergone of stability test (Fig. 5C).

Figure 5C shows VIS-NIR spectra of membranes, where a strong absorption in the near infrared wavelength region can be found at 800 nm beyond. NIR related peak position and intensity were observed remained consistent with each other, indicates stability of the mats under NIR exposure. The heating reproducibility and high photothermal stability of as-synthesized PDA modified PCL-DOX mats demonstrate the great potential as photothermal agents.

### Drug Release Study

**Figure 6:**
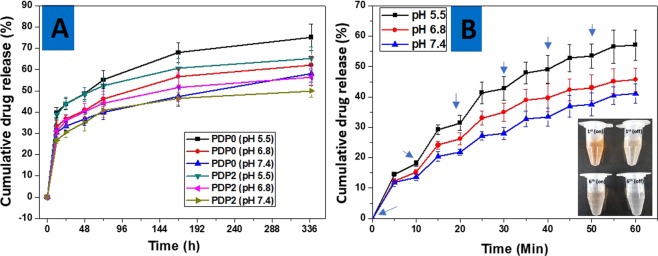
Drug release behavior. (**A**) DOX release profile from PCL-DOX (PDP0) and PCL-DOX/PDA (PDP2) membranes in PBS solutions at different pH values, and (**B**) DOX release profiles from PDP2 membranes as a function of NIR on-off cycles. Arrows show the irradiated time points. Digital images in Figure B (inset) are the representative released solution in first and six cycles when conducted at pH 5.5 PBS solution. Here, Data are expressed as mean values ± standard error (n = 3).

DOX was chosen as a model drug for investigating the drug release ability of electrospun PCL membrane coated with PDA since it has a broad-spectrum anticancer activity used in the treatment of a variety of solid tumors. The encapsulation efficiency of the DOX was obtained 90% and ~75% for PDP0 and PDP2 fibers. Figure 6A shows the cumulative drug release (%) from PDP0 and PDP2fibers into three different release media having different pH values; pH = 5.5, 6.8 and 7.4. Both fibers showed the drug released in a biphasic profile with initial burst release, which continued for 24 h (30–40%), the following time interval showed a slow and prolonged release at all pH conditions. Burst drug releases from the fibers are attributed to the quickly shedding of the drug that remained on the surface of the fiber. The results exhibited there was increased release was observed in PCL-DOX than PDP2in all time intervals in all pH conditions. For example, PDP0 showed 68.0, 56.7, 47.3% cumulative release in pH 5.5, 6.8 and 7.4 during 1-week incubation, respectively. However, DOX released was substantially decreased with PDA coating, reached 60.5, 51.7 and 46.4% in pH 5.5, 6.8 and 7.4, respectively in the same period (Fig. 6A). This is obvious, PDA coating prevents the drug release from initially because it acts as a barrier between medium and drug. Moreover, a released drug from the PCL-DOX fiber can be adsorbed by the PDA layer. The aromatic compound can interact with the conjugated polymer via π–π interaction^56^. Results further indicate the solution pH has influenced the release kinetics, the release was observed faster in a low pH in both PDP0 and PDP2 fibers. The solubility of DOX is improved with decreasing pH of the solution due to protonation of -NH_2_ groups on the DOX^57^, thereby increasing release was obviously observed higher in low pH. DOX release was continued 2 weeks period reaching the highest 75% to PDP0 at pH 5.5 which is 13 and 17% higher than its counterpart fiber that has been tested at pH 6.8 and 7.4, respectively. In the same period (2 weeks) PDP2 in pH 7.4 showed the lowest value which is 16 and 7% lower than its counterpart mat that had conducted at pH 5.5 and 6.8, respectively (Fig. 6A). These results indicate clearly the drug release from both fibers coated and uncoated is dependent to the pH of the PBS solution. Increased drug release observed in PDA coated fibers in low pH solution compared to higher pH solution can be explained two reasons. First reason can be explained by pH-responsive nature of PDA. At low pH, the amine groups of PDA became protonated and the neutralization of the charges led to an absence or decrease in the ionic interactions with positively charged DOX which resulting in the abrupt release of DOX from the PDA coated fibers^15,16,58^. Another reason could be due to increased solubility of the DOX into the medium at low pH condition compared to high pH solution. Given the mild acidic extracellular microenvironment compared with normal tissue, this stimulus‐responsive release tendency could provide a beneficial platform in pH‐mediated anticancer chemotherapy.

### NIR-Responsive Drug Release Study

The effect of NIR on the PDA coated fibers on PBS of different pH conditions was studied. PDP0 has not shown any heating property, therefore, these fibers were excluded from NIR illumination. NIR laser of 808 nm was subjected to PDA coated mats for 5 min and allowed to subsequent cooling at next 5 min at room temperature, this cycle was repeated for 6 times. In each point, samples were checked for drug content via UV-VIS spectroscopy. As shown in Fig. 6B, PDA coated mats in all pH conditions exhibited DOX release in on-off basis, with burst release during NIR on-process followed by a negligible release in absence of NIR (NIR off). The drug release was observed continuously decreased in the following cycle (Fig. 6B). The solution pH-dependent release was observed according to Fig. 6B. PDA coated fibers displayed 57.1, 45.6 and 41.08% release in pH 5.5, 6.8 and 7.4, respectively. Digital images of the drug released in different cycles shown clearly support the NIR mediated on-off mechanism of drug release (Fig. 6B). Improved release in lower pH compared to higher pH is attributed to the increased dissociation of electrostatically bound DOX molecules from PDA due to protonation of the PDA^58^. A clear acceleration of the drug release under NIR irradiation could be attributed to the local temperature increase due to the heating effect of PDA. Thus, increased temperature can affect the PCL layer to squeeze the drug molecules due to decreasing its drug holding capabilities by weakening the drug PCL interaction. The result clearly presents the NIR triggered drug delivery functionalities on an on-demand basis. Tuning of controlled drug release profile is a crucial aspect in the treatment of diseases. Precise and accurate delivery of the drug into the diseased site is important task in cancer treatment because it reduces the inevitable adverse effects of chemo which has a narrow therapeutic window.

### *In Vitro* Photothermal Treatment

**Figure 7:**
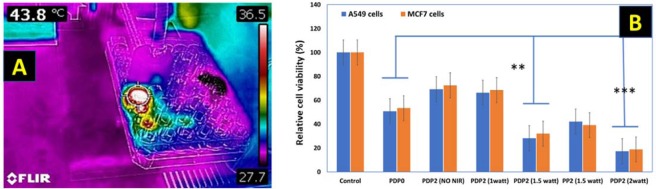
(**A**) Thermographic image of the cell treatment by NIR device, and (**B**) Cytotoxic effect of the samples against A549 and MCF7 cells determined by CCK-8 assay. Data are presented as mean values ± standard deviation (n = 3).

To evaluate the photothermal-chemo therapy effect, the different cells were incubated with PDP2 fibrous mats and subjected to 808 nm NIR laser irradiation as displayed in Fig. 7A. The CCK-8 assay was employed to determine the cell viability. As shown in Fig. 7B, PDP2 mat showed mild cytotoxicity (70–80%) without NIR irradiation while same composite material showed the increased cytotoxicity on the applied NIR density-dependent manner. When applied NIR at more than 1.5 W/cm^2^, cell viability was reduced significantly to cells that irradiated with 1 W/cm^2^ or not irradiated mats (Fig. 7B).

**Figure 8:**
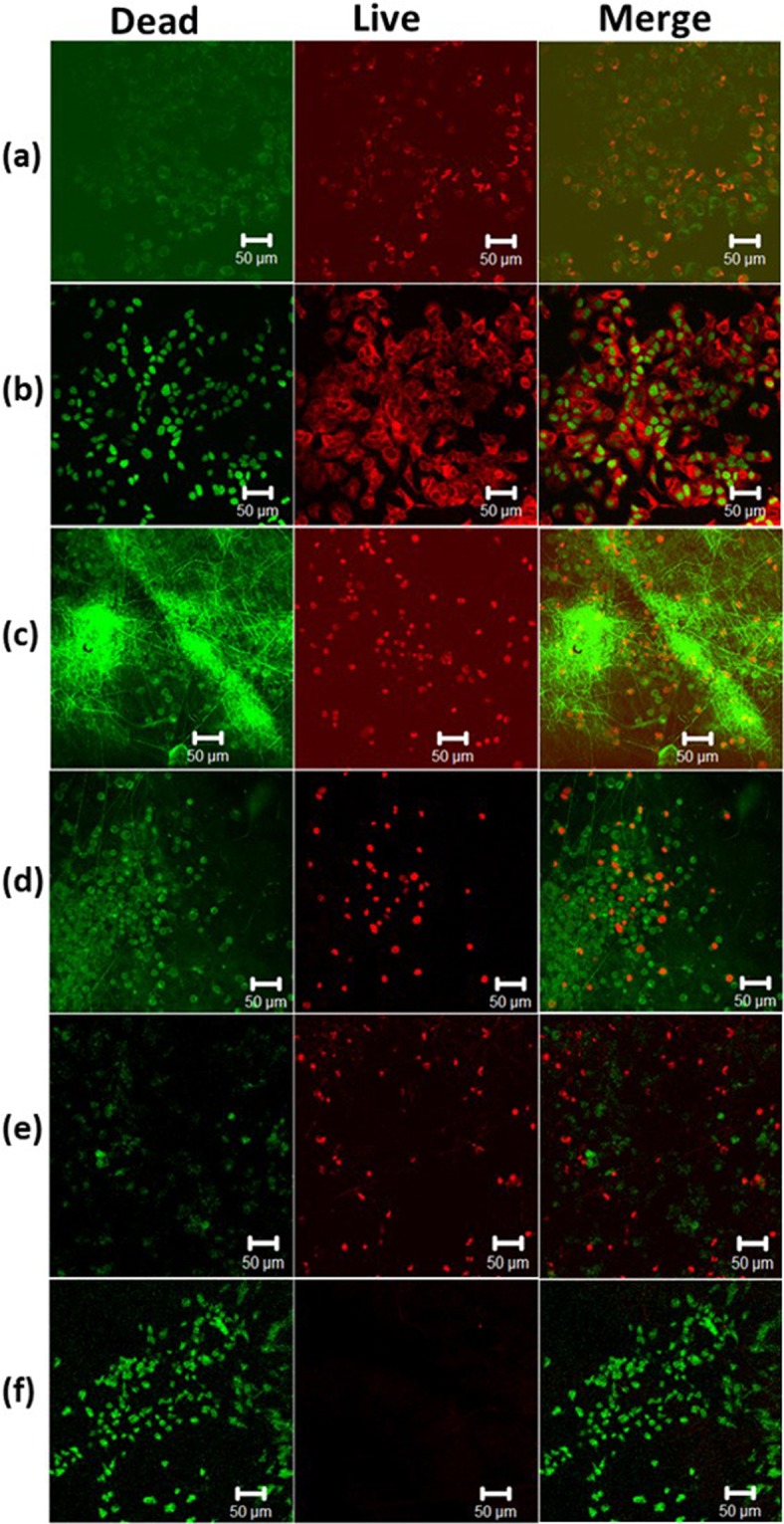
Confocal microscopy images of resazurin and SYTOX green stained MCF7 cells cultured on different membranes (**a**) PDP0, (**b**) PDP2 (without NIR), (**c**) PDP2 (1 W/cm^2^), (**d**) PDP2 (1.5 W/cm^2^), (**e**) PP2 (1.5 W/cm^2^) and (**f**) PDP2 (2 W/cm^2^). Resazurin stains live cells red while SYTOX stains dead cells green.

**Figure 9:**
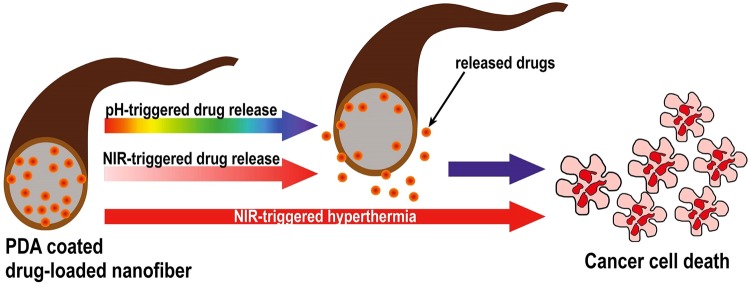
Schematic representation of cell death mechanism.

Similarly, PDP0 exhibited a significant reduction of cell viability compared to not irradiated PDP2 mats or PDP2 mats with irradiation by 1 W/cm^2^ (Fig. 7B). This result is accompanied by the burst release of DOX from PDP0 fiber into the cell medium, which directly induced cell deaths while PDA coated mats not subjected to NIR resulted in no apparent toxicity which is attributed to the coating of the biocompatible, non-toxic PDA over the drug-loaded mats. PDA coating prevents the DOX diffusion into medium somehow thereby lower toxicity in absence of external NIR. However, increased NIR exposure leads to significantly reduced cell viability compared to uncoated fiber is attributed to the synergistic effect of NIR itself which induced the hyperthermia, and NIR triggered increased drug release effect. NIR produces the local hyperthermia of ~45 and >50 °C for PDA coated mats irradiated with 1.5 and 2 W/cm^2^, respectively. Tumor cells are highly vulnerable to the temperature higher than 42 °C, and cells undergo ablation at that temperature. The chemo-photothermal therapy effect of PDP2 was further investigated taking the PCL/PDA fiber without DOX (PP2). Results exhibited NIR irradiation exhibited remarkably reduced the cell viability for both cells type (Fig. 7B). This shows sufficient temperature induced by NIR itself can have cytotoxicity but notably, cell viability was observed significantly lower than the PDP2 in presence of NIR (1.5 W/cm^2^). This result suggests the synergistic approach can lead to higher toxicity^16,59^. Furthermore, results of CCK assay was further counterchecked with the live and dead assay. Confocal imaging technique was used for the evaluation of live and dead MCF7 cells. Figure 8 shows the live cells are displayed in green while dead cells are in red. The microscopy observations revealed consistent with the result of CCK assay. Red cells are observed increasing with the increasing NIR laser power in NIR treated PDP2. The highest input of NIR laser resulted in the cells deaths almost completely. PDA has a high heating ability under the exposure to NIR. The mechanism of cell death via the synergistic effect of NIR mediated drug release and hyperthermia is proposed in schematically (Fig. 9).

The half maximum inhibitory concentration (IC50) value is the therapeutic concentration of drug which causes 50% cancer cell mortality^59,60^. Table 3 shows the comparison of the IC50 values for different materials. As shown in Table 3, the IC50 value of free DOX was found 1.66 ± 0.18 µm which is lower compared to the values 1.98 ± 0.13 µm and 2.96 ± 0.42 µm obtained for PDP0 and PDP2, respectively. However, the application of the NIR onto PDP2 mats showed the significantly lower IC50 values than those of PDP2. The reduction of the IC50 values was found dependently on irradiated NIR dose (Table 3). Induction of local hyperthermia due to NIR exposure of PDP2 mats and enhanced drug release due to hyperthermia synergistically could provide PDP2 mats with NIR irradiation with much lower IC50 value than the PDP2 mats not irradiated with NIR or PDP0 samples. In overall, *in-vitro* cells results indicated that PDA decoration into the drug-loaded fibers could be an effective localized platform that can inhibit tumor cell growth and eventual killing by delivering both a hyperthermia and heat mediated drug delivery under NIR exposure.

**Table 3:** IC50 values of different samples.

Materials	NIR dose (W/cm^2^)	IC50 value (µm)
Free DOX	0	1.66 ± 0.18
PDP0	0	1.98 ± 0.13
PDP2	0	2.96 ± 0.42
PDP2	1	1.61 ± 0.10
PDP2	2	0.43 ± 0.04

## Conclusions

We have reported the successful fabrication of localized anticancer drug delivery platform with bimodal functionalities. Facile electrospinning was used to fabricate PCL-DOX fiber and subsequent surface modification was performed by chemical polymerization of dopamine in various concentration. FESEM images confirmed the thin coating of the PCL-DOX membranes with extra nodules like PDA particles on the fiber surface. Resulting membranes exhibited the excellent photothermal behavior and stability in response to NIR laser of 808 nm. Similarly, PDA coated mats have shown improved DOX release at pH 5.5 than to higher pH solution (6.8 and 7.4). In addition, stimulation of PDA coated fibers by NIR light (808 nm, 1.5 W/cm^2^, 5 min), the amount of DOX released in the local environment demonstrated the on-demand delivery of cancer drugs. Evaluation of the anticancer effect of PDA coated membranes showed superior toxicity in response to NIR illumination in compared to mats not irradiated, could be attributed to the synergistic effect of NIR driven photothermal therapy and improved DOX release simultaneously. This study provides a promising composite material, which can be used in the localized treatment of cancers and other diseases with tunable drug releasing properties.
